# Exploring Two Pandemics in Academic Arena: Physical Activity and Sedentary Behaviors Profile of University Students in Bangladesh

**DOI:** 10.3390/ejihpe11020027

**Published:** 2021-04-16

**Authors:** Khalidur Rahman, Matteo Vandoni, Boris Cheval, Md Asaduzzaman, Mohammad Nayeem Hasan, Sabbir Tahmidur Rahman

**Affiliations:** 1 Department of Statistics, Shahjalal University of Science and Technology, Sylhet 3114, Bangladesh; nayeem5847@gmail.com (M.N.H.); sabbi_01@yahoo.com (S.T.R.); 2 Laboratory of Adapted Motor Activity (LAMA), Department of Public Health, Experimental and Forensic Medicine, University of Pavia, Via Forlanini 2, 27100 Pavia, Italy; vanmat06@unipv.it; 3 Swiss Center for Affective Sciences, University of Geneva, 1205 Geneva, Switzerland; boris.cheval@unige.ch; 4 Laboratory for the Study of Emotion Elicitation and Expression (E3lab), Department of Psychology, University of Geneva, 1205 Geneva, Switzerland; 5 Assistant Registrar (Hematology), Sylhet MAG Osmani Medical College Hospital, Sylhet 3100, Bangladesh; asad_somc@yahoo.com

**Keywords:** physical inactivity, COVID-19, pandemic, International Physical Activity Questionnaire (IPAQ), Exercise Self-Regulation Questionnaire (SRQ-E), university students

## Abstract

The overlay of the COVID-19 pandemic on the pandemic of physical inactivity has become a great concern. Both types of pandemics can decrease the health protection capacity and consequently increase complexity in human lives. This cross-sectional study intended to examine changes in physical activity and sedentary behaviors during the COVID-19 pandemic among university students in a second-tier city of Bangladesh. Two hundred and nine students responded to an online questionnaire administered via Google Survey. In addition to descriptive statistics, parametric and non-parametric tests for comparing means, medians and distributions were used to assess differences in activity traits before and during the COVID-19 pandemic. Results show that the occurrence of COVID-19 has significantly reduced the practice of walking and physical activities among the students. They are commonly motivated by introjected regulation. Father’s occupation and the type of family of a student have significant influences on the total physical activity in either situation. Bangladeshi university students have, particularly, been perceived as not generally used to vigorous physical activities. They are inactive compared to students from other countries. Thus, the public health policymakers and the corresponding authority should inspire the students to be more physically active by implementing different strategies such as increasing bicycling and walking facilities on the campus.

## 1. Introduction

Although the COVID-19 pandemic is termed as “black swan”—an unexpected event that changes everything [1]—another pandemic of distinct nature has already been prevailing over the world for years, and it is known as the pandemic of physical inactivity [2]: the fourth leading cause of global mortality due to stimulated non-communicable diseases. Physical inactivity is held responsible for an estimated 3.2 million deaths per year [3]. This global pandemic of physical inactivity is persisting owing to the fact that 16.6% to 34.5% of adults are not physically active [4]. In this regard, it is to be noted that the inactivity level among Bangladeshi adults is 34.5% of this 37.7% and 31.6% are from urban and rural areas respectively [5]. The percentage is higher among university students worldwide as they spend numerous hours on study-related sitting or online social media. Thus a significant portion of them leads a sedentary lifestyle [6]. However, the self-determination level of an individual may play a significant role in the adaptation and care of health-promoting behaviors like physical activity [7]. Currently, the merging of the COVID-19 pandemic with the pandemic of physical inactivity is further reducing physical activity and dramatically increasing sedentary behaviors [8]. This is problematic because physical activity is associated with many beneficial consequences related to physical and mental health [9,10]. Accordingly, the reduction of physical activity due to the COVID-19 pandemic can lead to a synergistic negative impact on individuals’ physical and mental health.

Thus, there is a need to evaluate the physical inactivity and sedentary behaviors among the university students that derive from the two pandemics. Such an evaluation will help the policymakers design an action plan to improve the conditions. To the best of our knowledge, although there are some studies documenting associations of the COVID-19 pandemic with physical inactivity and sedentary behaviors [8,11,12,13] and these studies have found that physical activity has deteriorated, particularly among university students [13,14,15], there has been no study to evaluate the effects of the COVID-19 pandemic on physical activity and sedentary behaviors among the university students in Bangladesh.

In the present study, we aim to investigate the effects of two stated pandemics on physical activity and sedentary behaviors predominating among students at a university located in a second-tier city of Bangladesh. Specifically, the present study aimed: (i) to quantify physical activity and sedentary behaviors among the selected university students and the corresponding differences and associations between the pre and during the COVID-19 pandemic situations, (ii) to examine the relationships of individual-level motivation from the perspective of self-determination theory, possible covariates and confounders with physical activity before and during the COVID-19 pandemic, (iii) to identify the separate and common factors that influence and link the physical activity in two pandemics and thus, the efforts need to be taken to get students physically active after COVID-19 pandemic.

## 2. Materials and Methods

### 2.1. Study Design and Participants

The current study is a cross-sectional investigation. A population-based sample of the students of Shahjalal University of Science and Technology (SUST) was selected to assess the students’ physical activity. The SUST is located in the second-tier city of Sylhet. Two hundred and nine (at least 30 in each semester) young students have participated from this university to provide the relevant data. In a previous study [16], an intraclass correlation coefficient based on socio-demographic covariates was found to be around 0.7 among groups of university students in terms of different commuting modes; thus, the following formula [17] has been used to calculate the minimum size of the sample:

E=1mk21−ρ^21+k−1ρ^2kk−1m

where, *k* = the required sample size in each group, *m* = number of groups = 6 *Z_α_*_/2_ = the *z* score from standardized normal distribution at α% level of significance = 1.96 (with 5% significance level), $\hat{\rho }$ = the estimated intraclass correlation coefficient = 0.7, and *E* = the margin of error = 0.05 (5% margin of error). So, *k* = 7 (approx.). Hence, this study has used more than the minimum required sample for analysis.

Although there is no formal committee at SUST to approve the research’s ethical issues, verbal approval was taken from the corresponding authority.

### 2.2. Instruments

The list of confounders, covariates, and targeted variables along with the related measurement tools, has been described below.

Per-capita household income is one of the common confounders and is related to physical activity and physical fitness. Thus, for each student, information on family income has also been collected.

Socio-demographic covariates comprise running semesters, age, sex, educational qualification and occupation of parents, type of family, residential places, body mass index (BMI), chronic condition (e.g., asthma, coronary artery disease etc.) (yes vs. no), and COVID-19 symptoms (yes vs. no). Furthermore, in addition to demotivation, each student might either be intrinsically or extrinsically motivated for physical activity and such assessments were done by utilizing the Exercise Self-Regulation Questionnaire (SRQ-E) based on self-determination theory [18,19]. All the items of the original SRQ-E were considered. Accordingly, the test–retest reliability for each of its four subscales (external regulation, introjected regulation, identified regulation, and intrinsic motivation) have also been measured to assess if the participants’ Likert scale scores remain consistent. In addition, the Relative Autonomy Index (RAI) for each student has been computed by using the weighted average of the responses to each of the subscale’s items and the formula used as 2 × intrinsic + identified − introjected − 2 × external. In this formula, positive weights are assigned to the autonomous subscales and negative weights are assigned to the controlled subscales [20,21].

The health-related physical activity and sedentary behaviors of each student over the usual 7 days before the COVID-19 pandemic and last 7 days during the COVID-19 pandemic were surveyed using the long form of the self-administered International Physical Activity Questionnaire (IPAQ) [22]. This questionnaire aids in assessing the hours and minutes of physical activity and sedentary behaviors per week under the five domains. Any designated activity lasting for more than 10 min has been considered for inclusion. Since a person usually spends around 8 h in a day sleeping, the students whose total activities in all domains exceed 6720 min per week in either before or during the COVID-19 should be excluded from the study. Similarly, those students also need not be considered for investigation who delineate no activity throughout all domains (which is totally unusual) in any period. Besides, if the total of any physical activity of walking, as well as exercise of moderate-intensity and vigorous-intensity is greater than 3 h/day, it should be truncated to 3 h. The participating students living in different parts of Bangladesh and confined in houses have responded to an online questionnaire.

MET means metabolic equivalent, and it is used to measure the energy level (oxygen consumption) needed for a person to perform a particular activity. One unit of MET is equivalent to 1 kcal/kg/h; that is, the energy level at resting or sitting still. According to the prescribed protocol [23], the following values of MET were used to score the disclosed physical activities: moderate activities inside the home = 3.0 METs, walking at work/for transport/in leisure = 3.3 METs, moderate activities at work/in the garden or yard/in leisure = 4.0 METs, vigorous activities in the garden or yard = 5.5 METs, cycling for transport = 6.0 METs and vigorous activities at work/ in leisure = 8.0 METs. Scores for all physical activities and time for sedentary behaviors were calculated, and these are consistent with the procedures for data processing and analysis of the IPAQ.

### 2.3. Procedure

A combined questionnaire was developed and it had four parts. The first part related to sociodemographic information of the students; the next two parts were related to their physical and sedentary behaviors before and during the COVID-19 pandemic, respectively, based on IPAQ. In the last part, using SRQ-E and the rating scale of 1 to 7, the students were asked to indicate how true each of the selected reasons is for why she or he is, or would like to be, active in terms of physical activity. The basic relevant data were collected in the first two weeks of June 2020 through the questionnaire in Google Survey. The participating students have given their consent by the online form.

### 2.4. Data Analysis

In addition to descriptive statistics of all the measured variables, parametric and non-parametric tests for comparing means, medians and distributions were used to assess differences in traits of physical activity before and during the COVID-19 pandemic. Exploratory Factor Analysis (EFA) has been used to find out the separate and common factors respectively that are allied with the two pandemics. Besides, for the analysis of reliability for each of the four subscales of SRQ-E, Cronbach’s Alpha was used.

## 3. Results

### 3.1. Data Cleaning

Eighteen students quantified the total activity that exceeded 16 h/day before and/or during the COVID-19 pandemic. Another 16 did not provide any activity at all in either of the study periods. Thus, the information of both these student bunches has been excluded from the study and a data set of 175 students is finally considered for analysis.

### 3.2. Summery of Covariates and Confounder

Information of all finally selected students on categorical covariates is summarized in Table S1 in supplemental materials.

Body Mass Index (BMI) has been calculated for each participant and found that 22 (12.57%), 116 (66.29%), 29 (16.57%), and 8 (4.57%) students have BMI less than 18.5, between 18.5 to 24.9, between 25 to 29.9, and greater than 30. These ranges are categorized in Section 3.3.2 as underweight, normal weight, overweight and obesity, respectively. Regarding SRQ-E, Cronbach’s alpha for its subscales external regulation, introjected regulation, identified regulation, and intrinsic motivation are 0.59, 0.65, 0.82 and 0.56, respectively. Low values of Cronbach’s alpha indicate that validity and reliability original SRQ-E should be further done for Bangladeshi students in a large-scale study. Calculated RAI for all students ranging from −9 to 13.8, 13 (7.43%) scored negative, 3 (1.71%) scored zero and the rest 159 (90.86%) scored positive values. The negative values and the zeros, as well as the positive values are also categorized in Section 3.3.2 as extrinsically motivated and intrinsically motivated to be active. The descriptive statistics of sociodemographic non-categorical covariates are given in Table S2 in supplemental materials.

Since family income as a confounder is a continuous variable and assumed to follow normal distribution, a parametric *t*-test-based means comparison before and during the COVID-19 pandemic is displayed in Table S3 in Supplemental Materials.

### 3.3. Total Physical Activity and Sedentary Behaviors

#### 3.3.1. Extraction of Total Scores in Physical Activities and Time in Sedentary Behaviors

The levels of MET minutes per week and minutes per week spending, separately, on different physical activities and sedentary behaviors under the five domains among the selected university students before and during the COVID-19 pandemic are compressed in Table 1. Given that the distribution of time and energy expenditure amongst various populations is non-normal [22], in addition to relevant descriptive statistics, non-parametric Wilcoxon signed-rank test for medians comparisons are presented in this table.

#### 3.3.2. Analysis in the Context of Covariates

The non-parametric Mann–Whitney U test has been used to test whether the distribution of total physical activity is identical or not through the two groups of a categorical variable. For the categorical variables with more than two groups, the appropriate non-parametric Kruskal–Wallis H test was applied. All the results are estimated and provided in Table 2. In Table 3, descriptive statistics for total physical activity are described for each group of categorical covariates that were found to yield statistically significant unequal distributions at a 10% level of significance.

The total physical activity levels of university students under the normal situation in different countries (which also include the result of the present study) are listed in Table 4.

### 3.4. Physical Activity and Its Domains

In Table 5, domain-specific descriptive statistics of physical activities and the subsequent comparisons are depicted. Again, as in Table 1, the non-parametric Wilcoxon signed-rank test for medians comparison is appropriate and has been applied.

**Figure 1:**
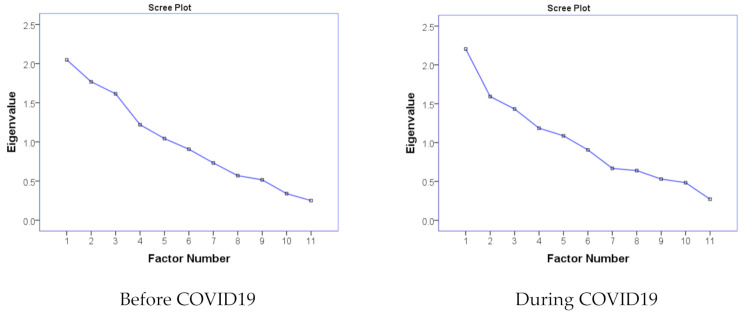
Scree Plots.

In this line, the results obtained from subdomains based on multivariate analysis will help to get further insight into the behavior of students for physical activities. Although the use of polychoric correlation is recommended to estimate the strength of the linear relationship between ordinal variables [31], the Karl Pearson correlation coefficient could be applied when the variables have more than five categories [32]. Thus, the Exploratory Factor Analysis (EFA) based on the correlation matrix of Pearson’s coefficients has been utilized in this study. The Principal Axis Factoring (PAF) extraction method along with the direct oblimin rotation technique has extracted five factors with eigenvalues >1 in each situation before and during COVID-19, and are presented in Table 6. The corresponding scree plots are also shown in Figure 1. As the number of factors can be chosen by combining with eigenvalues > 1 with the sharpest drop of the eigenvalue in the scree plot, three factors for before COVID-19 and only one factor for during COVID-19, exclusively, have yet to be recognized.

**Figure 2:**
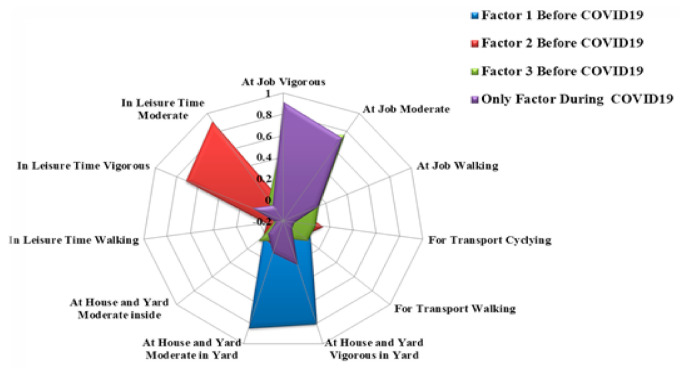
Rotated factor loadings of factors.

The radar charts, as shown in Figure 2, illustrate the loadings of the physical activities of three dominant factors before COVID-19 and the only one factor during COVID-19. Changes in any activity concerning factors are also visually depicted. From this figure, it is obvious that each factor is partially overlaid with the others.

## 4. Discussion

The quantification and evaluation of different physical activities and sedentary behaviors in students indicate that the prevalence of COVID-19 has significantly decayed the amount of walking, moderate and total physical activities. In contrast, sitting and sedentary behaviors have increased dramatically. Such changes may be caused, in addition to restrictions on movement, by the suspension of campus activities. Besides, the part-time jobs of tutoring students at home are not functioning during this pandemic. As the online classes were not introduced in June 2020, students might spend the bulk of their time on online social media. However, students generally refrain from vigorous physical activities in normal situations and during the period of COVID-19. This is a matter of concern and should be noted for taking the necessary next steps to improve habits [33]. The authority could, for instance, suggest the restriction on vehicles other than bicycles on the campus in the evening so students are bound to walk or use bicycles in their leisure time. They should also increase the number of available rented bicycles that are now supplied by Jobike. Separated and safe lanes for cycling and walking should be ensured.

The following factors have no significant impact on the total physical activity of the students before and during the COVID-19 pandemic: the semester in which a student is involved in academic activities, gender, educational and occupational level of their mother, chronic health problems, bearing the symptoms of COVID-19, and a motivated attitude for physical activity. Nonetheless, the occupational status of father and the type of family of a student have significant influences on total physical activity in both situations. Again, the effects of academic qualification of father and the residential place in Sylhet before the COVID-19 pandemic and area of original residence and BMI during the COVID-19 pandemic have, respectively, been noted. In the context of Bangladesh, the fathers who lead the life by self-employed agricultural jobs have a tendency to engage their successors in their works and thus, these successors are more active than other students. The offspring of a joint family have more physical activity than a nuclear family caused by the duties maintaining for the family. Furthermore, an educated father may be more conscious of the children to encourage physical activity under normal conditions. Students residing in messes are necessarily more active to fulfill their own needs. Under the present circumstance of COVID-19, the students are residing in their homes and those in rural areas have more opportunities for physical activity than those in urban settings. It has been discovered that obese students are more physically active. This may be for the reason that to reduce the comorbidity of the risk of COVID-19, such students have engaged themselves in more physical activity. This practice likely stems from the knowledge that physical activity reduces morbidity and mortality from COVID-19.

From the comparison of the level of total physical activity among the university students from different countries in a normal situation, it has been shown that the European students are more active than their counterparts in Asia and Africa [24,25,26,27,28,29,30]. The physical activity level of Bangladeshi students is closer to Indian and Chinese, but lower than them. This finding suggests that concerned policymakers should gear up and encourage the students to be more physically active. Otherwise, the predominance of non-communicable diseases cannot be controlled. The non-significant effect of gender on physical activity contradicts with the findings from other studies [26,30,34,35]. In addition, female students were found to be more physically active than males, which is unusual in other countries [26,30].

Domain-specific evaluation of physical activity further ensures that job-oriented activity has declined as a result of the incidence of COVID-19. Movement by transportation and activity generated in leisure have also been restricted in the current condition. However, it is rational that activity at home and yard should not be affected by the outbreak of this infectious disease.

A regulation that a person accepted but does not take as her/his own is termed as an introjected regulation. On the other hand, the value of the activity that a person treats as personally important is considered an identified regulation, and integrating that identification with other aspects is defined as integrated regulation [18]. The sign and size of loadings in the three identified factors in a normal situation and one factor under the prevalence of COVID-19 suggest that the physical activity of students is motivated and dominated by integrated regulation, identified regulation, and introjected regulation in a normal situation and only by introjected regulation in the presence of COVID-19. That is, university students are confined and have very limited facility in doing physical activity in the current pandemic.

Some limitations are present in this study. As this study was conducted during the COVID-19 pandemic, there was no option to conduct the survey at the de jure level. Internet data were sent to the invited students to attend the survey, but some could not participate due to the unavailability of a full Wi-Fi network. Although the study collected information from students living in different regions of Bangladesh, it would be more representative if the information could be collected from all of the universities of Bangladesh. In addition, students were required to provide information on walking, moderate-to-vigorous physical activities and sedentary behaviors over a period of 7 days in normal conditions. As they did so by recalling the earlier activities, the data may suffer from a lack of accuracy.

## 5. Conclusions

In this study, the physical activities and sedentary behaviors of the students from a second-tier city in Bangladesh were evaluated to identify their performances in normal conditions and the COVID-19 pandemic, respectively. The assumed covariates and confounder were also examined to assess their link with activities.

The study reveals that the prevalence of COVID-19 has significantly hindered walking, moderate and total physical activities among the students while sitting and sedentary behaviors have increased dramatically. In both situations, however, students are commonly motivated by introjected regulation. Bangladeshi university students are too physically inactive compared to students in Western countries. They are even less active than students in neighbouring countries. It is inspiring to observe that female students are more active than their male counterparts. Most of the students were found to be intrinsically motivated to be physically active. However, vigorous physical activities are almost absent among them in any situation.

## Figures and Tables

**Table 1:** Comparison of Physical Activity and Sedentary Behaviors in Two Situations.

Activity	Situation	Range		Inter Quartile Range		Median	Skewness	*p*-Value
		High	Low	Q3	Q1			
Walking (MET-minutes/week)	B	4851.0	0.0	1650.0	495.0	825.0	1.47	<0.001
	D	4158.0	0.0	660.0	99.0	346.5	2.77	
Moderate (MET-minutes/week)	B	13,320.0	0.0	1650.0	60.0	480.0	2.67	<0.001
	D	5400.0	0.0	954.0	40.0	287.5	2.21	
Vigorous (MET-minutes/week)	B	7200.0	0.0	480.0	0.0	0.0	3.00	<0.001
	D	2520.0	0.0	0.0	0.0	0.0	2.79	
Total Physical Activity (MET-minutes/week)	B	17,052.0	0.0	4146.0	927.0	2148.0	1.79	<0.001
	D	8238.0	0.0	2271.0	388.5	891.0	1.56	
Total Sitting—Including Transport (Minutes/week)	B	5040.0	0.0	2960.0	1110.0	1590.0	0.68	<0.001
	D	6720.0	0.0	3360.0	1400.0	2100.0	0.85	

B = Before COVID-19, D = During COVID-19, Q3 = Third Quartile, Q1 = First Quartile.

**Table 2:** Comparison of the Distributions of Total Physical Activity (MET-Minutes/Week) by Categorical Characteristics.

Characteristics	Categories	*p*-Value for B	*p*-Value D
Semester	First Year First Semester	0.116	0.102
	Second Year First Semester		
	Third Year First Semester		
	Fourth Year First Semester		
	Masters First Semester		
	Masters Second Semester		
Gender	Male	0.779	0.148
	Female		
Father’s Education	Below Secondary	0.091 **	0.860
	Secondary or Above		
Mother’s Education	Below Secondary	0.367	0.811
	Secondary or Above		
Father’s Occupation	Agri Worker	0.006*	0.071 **
	Job/Service		
	Non-Agriculture Worker		
	Others		
Mother’s Occupation	Housewife	0.106	0.842
	Job/Service		
Type of Family	Joint	0.022 *	0.043 *
	Nuclear		
Place of Residence in Sylhet	Hall	0.068 **	0.104
	Own Residence		
	Student Mess		
Area of Residence	Rural	0.406	0.008 *
	Urban		
Chronic Complications	No	0.873	0.857
	Yes		
Symptoms of COVID-19	No	0.483	0.381
	Yes		
BMI	Underweight	0.107	0.092 **
	Normal Weight		
	Overweight		
	Obesity		
RAI	Extrinsically motivated	0.275	0.953
	Intrinsically motivated		

B = Before COVID-9, D = During COVI-19, * Significant variation at *p* < 0.05, ** Significant variation at *p* < 0.10.

**Table 3:** Descriptive statistics for Total Physical Activity Corresponding to Significant Categorical Characteristics.

Characteristics	Situation	Categories	Range		Inter Quartile Range		Median
			High	Low	Q3	Q1	
Father’s Education	B	Below Secondary	9235.0	264.0	4042.5	387.75	1205.0
		Secondary or Above	17,052.0	0.0	4158.0	1033.13	2243.5
Father’s Occupation	B	Agri Worker	9235.5	330.0	6202.5	1669.8	2831.5
		Job/Service	17,052.0	148.5	6204.0	1155.0	2706.0
		Non-Agriculture Worker	5100.0	379.5	3079.9	594.0	1213.8
		Others	13,683.0	0.0	3219.0	773.6	1639.3
	D	Agri Worker	8238.0	80.0	4084.6	911.0	2929.0
		Job/Service	6555.0	0.0	2430.3	412.5	858.0
		Non-Agriculture Worker	3093.0	99.0	1022.6	379.5	800.5
		Others	6048.0	0.0	2018.8	193.5	878.8
Type of Family	B	Joint	17,052.0	270.6	7257.0	1398.0	3823.5
		Nuclear	14,013.0	0.0	3807.0	891.0	1995.5
	D	Joint	6555.0	0.0	3204.8	522.0	1965.0
		Nuclear	8238.0	0.0	2045.0	318.0	866.5
Place of Residence in Sylhet	B	Hall	12,357.0	226.0	3885.8	1159.0	2010.3
		Own Residence	6291.0	231.0	2946.4	643.5	1177.3
		Student Mess	17,052.0	0.0	4621.0	1017.8	2408.0
Area of Residence	D	Rural	8238.0	60.0	3357.8	455.3	1219.3
		Urban	5574.0	0.0	1619.0	328.0	792.0
BMI	D	Underweight	4254.0	99.0	952.5	273.0	476.8
		Normal Weight	8238.0	0.0	2385.0	340.1	910.0
		Overweight	4188.0	90.0	2310.5	504.5	891.0
		Obesity	5701.0	132.0	3340.5	558.5	1758.0

B = Before COVID-19, D = During COVID-19, Q3 = Third Quartile, Q1 = First Quartile.

**Table 4:** Level of Physical Activity among University Students in Different Countries.

Continent	Country	Article	Total Physical Activity(MET-Minutes/Week)
Africa	Egypt	[24]	Median = 2256
	Nigeria	[25]	Mean = 4449.92, Median = 3999.75
Asia	Bangladesh		Median = 2148.0 (Combined), Mean = 3161.36 (Combined) Median = 2086.75 (Male), Median = 2148 (Female)
	China	[26]	Median = 2274 (Male), Median = 1504 (Female)
	India	[27]	Median = 2347.8
Europe	Poland	[28]	Mean = 5954, Median = 5289
	Romania	[29]	Mean = 5343.92
	Turkey	[28]	Mean = 3095, Median = 2772
	Ukraine	[30]	Mean = 4233.4
	Visegrád countries	[30]	Mean = 5588.5

**Table 5:** Level of Physical Activity under Different in Two Situations.

Domains	Situation	Range		Inter Quartile Range		Median	Skewness	*p*-Value
		High	Low	Q3	Q1			
At Job (MET-minutes/week)	B	10,986.0	0.0	636.0	0.0	0.0	3.17	<0.001
	D	2820.0	0.0	0.0	0.0	0.0	4.12	
For Transport (MET-minutes/week)	B	7812.0	0.0	1386.0	330.0	594.0	2.53	<0.001
	D	4074.0	0.0	396.0	0.0	99.0	4.17	
At House and Yard (MET-minutes/week)	B	13,320.0	0.0	875.0	0.0	285.0	4.56	0.097
	D	5000.0	0.0	720.0	0.0	210.0	2.51	
In Leisure Time(MET-minutes/week)	B	12,090.0	0.0	933.0	0.0	420.0	5.24	0.010
	D	4878.0	0.0	693.0	0.0	179.0	2.57	

B = Before COVID19, D = During COVID19, Q3 = Third Quartile, Q1 = First Quartile.

**Table 6:** Rotated Factor Loadings and Variance Explained for Physical Activity.

Domains	Subdomains	Situation									
		Factor in B					Factor in D				
		1	2	3	4	5	1	2	3	4	5
At Job	Vigorous	−0.025	−0.030	0.608	0.002	−0.021	0.916	0.072	−0.078	0.053	0.081
	Moderate	−0.002	−0.017	0.761	−0.030	0.099	0.725	0.021	0.157	−0.155	−0.021
	Walking	−0.018	−0.011	0.128	0.599	−0.107	0.121	−0.084	0.000	−0.559	−0.058
For Transport	Cycling	0.014	0.140	0.077	0.172	−0.154	−0.109	0.187	0.014	−0.716	0.038
	Walking	0.108	0.012	0.075	0.446	0.108	−0.101	0.468	−0.026	−0.191	0.089
At House and Yard	Vigorous in Yard	0.803	−0.051	−0.029	−0.031	−0.142	0.224	0.587	0.055	0.025	−0.056
	Moderate in Yard	0.845	0.049	−0.010	0.030	0.285	0.110	0.277	0.790	0.058	−0.198
	Moderate inside	0.030	0.032	0.080	0.069	0.601	−0.026	−0.113	0.557	−0.036	0.141
In Leisure Time	Walking	−0.058	−0.055	−0.115	0.567	0.048	−0.135	0.345	−0.017	0.056	0.537
	Vigorous	−0.018	0.713	−0.093	−0.067	0.028	0.098	−0.010	−0.052	−0.038	0.202
	Moderate	−0.010	0.915	0.039	−0.012	0.026	−0.021	−0.047	0.102	0.034	0.447
	Variance explained (%)	14.74	12.66	11.11	5.65	3.69	16.61	9.76	9.17	5.62	3.84
	Cumulative variance (%)	14.74	27.40	38.52	44.17	47.86	16.61	26.37	35.54	41.15	44.99

B = Before COVID19, D = During COVID19, Q3 = Third Quartile, Q1 = First Quartile.

## Data Availability

Not applicable.

## Supplementary Materials

The following are available online at https://www.mdpi.com/article/10.3390/ejihpe11020027/s1. Table S1: Frequency Distributions by Categorical Characteristics. Table S2: Descriptive Statistics of Non-Categorical Characteristics. Table S3: Family Income (in Taka) of Students in Two Situations.
