# Supplementary material for: Exploring Two Pandemics in Academic Arena: Physical Activity and Sedentary Behaviors Profile of University Students in Bangladesh
Source: Eur J Investig Health Psychol Educ. 2021 Apr 16;11(2):358–71. doi: 10.3390/ejihpe11020027 (PMC8314348; doi:10.3390/ejihpe11020027)
Supplement: Supplementary file 1 [file ejihpe-11-00027-s001.zip › ejihpe-1140441-supplementary.pdf]

Table S1: Frequency Distributions by Categorical Characteristics

Characteristics	Frequency	Percent
**Semester**		
First Year First Semester	30	17.1
Second Year First Semester	28	16.0
Third Year First Semester	29	16.6
Fourth Year First Semester	34	19.4
Masters First Semester	27	15.4
Masters Second Semester	27	15.4
**Gender**		
Male	120	68.6
Female	55	31.4
**Father's Education**		
Below Secondary	21	12.0
Secondary or Above	154	88.0
**Mother's Education**		
Below Secondary	24	13.7
Secondary or Above	151	86.3
**Father's Occupation**		
Agri Worker	10	5.7
Job/Service	81	46.3
Non-Agriculture Worker	18	10.3
Others	66	37.7
**Mother's Occupation**		
Housewife	159	90.9
Job/Service	16	9.1
**Type of Family**		
Joint	28	13.7
Nuclear	151	86.3
**Place of Residence in Sylhet**		
Hall	40	22.9
Own Residence	30	17.1
Student Mess	105	60.0
**Area of Residence**		
Rural	58	33.1
Urban	117	66.9
**Chronic Complications**		
No	155	88.6
Yes	20	11.4
**Symptoms of COVID-19**		
No	164	93.7
Yes	11	6.3

Table S2: Descriptive Statistics of Non-Categorical Characteristics

**Characteristics**	**Mean (SD)**
Age (in Years)	21.92 (1.75)
Height (in Meters)	1.67 (0.10)
Weight (in Kilograms)	63.38 (12.51)
BMI (kg/m^2)	22.26 (3.36)
RAI	6.19(4.10)

SD = Standard Deviation.

Table S3: Family Income (in Taka) of Students in Two Situations

<table>
<tr><th rowspan="2">Situation</th><th rowspan="2">Mean</th><th rowspan="2">SD</th><th colspan="2">Range</th><th colspan="2">Difference</th><th rowspan="2">Correlation</th><th rowspan="2">p-value</th></tr>
<tr><td>High</td><td>Low</td><td>Mean</td><td>SD</td></tr>
<tr><td>B</td><td>47574.71</td><td>51897.66</td><td>600000</td><td>3000</td><td rowspan="2">13387.93</td><td rowspan="2">22215.19</td><td rowspan="2">0.91</td><td rowspan="2"><.001</td></tr>
<tr><td>D</td><td>34186.78</td><td>3358.82</td><td>500000</td><td>0.00</td></tr>
</table>

SD = Standard Deviation, B = Before COVID-19, D = During COVID-19.
